# Biomechanical testing of a polymer-based biomaterial for the restoration of spinal stability after nucleotomy

**DOI:** 10.1186/1749-799X-4-25

**Published:** 2009-07-15

**Authors:** Aldemar A Hegewald, Sven Knecht, Daniel Baumgartner, Hans Gerber, Michaela Endres, Christian Kaps, Edgar Stüssi, Claudius Thomé

**Affiliations:** 1 Department of Neurosurgery, Medical Faculty Mannheim, University of Heidelberg, Theodor-Kutzer-Ufer 1-3, 68167 Mannheim, Germany; 2 Institute for Biomechanics, ETH Zurich, Wolfgang Pauli-Str. 10, 8093 Zürich, Switzerland; 3 Tissue Engineering Laboratory, Department of Rheumatology, Charité – University Medicine Berlin, Tucholskystrasse 2, 10117 Berlin, Germany; 4 TransTissue Technologies GmbH, Tucholskystrasse 2, 10117 Berlin, Germany

## Abstract

**Background:**

Surgery for disc herniations can be complicated by two major problems: painful degeneration of the spinal segment and re-herniation. Therefore, we examined an absorbable poly-glycolic acid (PGA) biomaterial, which was lyophilized with hyaluronic acid (HA), for its utility to (a) re-establish spinal stability and to (b) seal annulus fibrosus defects. The biomechanical properties range of motion (ROM), neutral zone (NZ) and a potential annulus sealing capacity were investigated.

**Methods:**

Seven bovine, lumbar spinal units were tested in vitro for ROM and NZ in three consecutive stages: (a) intact, (b) following nucleotomy and (c) after insertion of a PGA/HA nucleus-implant. For biomechanical testing, spinal units were mounted on a loading-simulator for spines. In three cycles, axial loading was applied in an excentric mode with 0.5 Nm steps until an applied moment of ± 7.5 Nm was achieved in flexion/extension. ROM and NZ were assessed. These tests were performed without and with annulus sealing by sewing a PGA/HA annulus-implant into the annulus defect.

**Results:**

Spinal stability was significantly impaired after nucleotomy (p < 0.001). Intradiscal implantation of a PGA-HA nucleus-implant, however, restored spinal stability (p < 0.003). There was no statistical difference between the stability provided by the nucleus-implant and the intact stage regarding flexion/extension movements (p = 0.209). During the testing sequences, herniation of biomaterial through the annulus defect into the spinal canal regularly occurred, resulting in compression of neural elements. Sewing a PGA/HA annulus-implant into the annulus defect, however, effectively prevented herniation.

**Conclusion:**

PGA/HA biomaterial seems to be well suited for cell-free and cell-based regenerative treatment strategies in spinal surgery. Its abilities to restore spinal stability and potentially close annulus defects open up new vistas for regenerative approaches to treat intervertebral disc degeneration and for preventing implant herniation.

## Background

When implementing regenerative strategies to treat degenerative spinal diseases, we have to keep in mind that, ultimately, our main objective is not tissue regeneration, but the elimination of pain for the patient. In this context, it is useful to differentiate between preventive and curative treatment approaches [16]. Hence, each approach has its particular clinical indications and needs to address specific disease-related problems in order to be beneficial for the patient.

In spinal surgery, preventive procedures to avoid common follow-up complications are conceivable in operations for disc herniation with predominant radicular leg pain. Here, surgery is done for neural decompression. The initial success rate is high: up to 92% report a good or excellent outcome after 4 to 6 months following surgery [34]. But in the long-term follow-up, intervertebral disc herniations can be complicated by two major problems: (a) painful de-generation of the spinal segment [23,37,24,4,5], and (b) re-herniation [10,5,6]. Thus, it is reasonable to think about possible interventions during primary surgery to avoid these complications.

Taking a closer look at these complications, painful degeneration of the spinal segment after surgery for intervertebral disc herniation can be found in up to 47% of the patients after 2 years follow-up and correlates with pathological modic changes in the adjacent vertebral bodies [5,6]. Atlas et al. reported that after 10 years, 31% complain about back pain with the same intensity or worse than shortly before operation [4]. In the literature, three major causes for the development of pain are suggested: (1) segmental spinal instabilities and pathological loading patterns, caused by preceding degeneration and the operation itself [26,8,21,33,28,31,3], (2) pathological ingrowths of nerves into the inner layers of the torn annulus fibrosus, sometimes even penetrating the nucleus pulposus [13,29,30] and (3) pain-mediating inflammatory cytokines like TNF-alpha and IL-1 secreted by disc cells and granulation tissue [9,25].

Re-herniation occurs predominately within the first two years after surgery. From our own data, we can report a re-operation rate of approximately 10% after a mean of 9 months because of re-herniation [5]. Moreover, re-operation rates up to 21% have been reported with annulus fibrosus defects larger than 6 mm [10]. Especially in the context of nucleus implants, defects larger than 6 mm will regularly occur due to access-related enlargement of the defect and post a considerable safety problem.

Current regenerative approaches for the biological repair of intervertebral disc tissue to prevent painful degeneration of the spinal segment focus on the transplantation of culture-expanded, autologous, disc-derived cells. A first clinical trial indicates that this approach reduces back pain and may prevent loss of disc height [22]. More advanced tissue engineering approaches focus on the use of absorbable biomaterials combined with autologous cells and/or bioactive factors [16]. The use of biomaterials potentially improves biomechanical properties, allows even distribution of cells and may guide tissue formation and regeneration [32].

Recently, it has been shown that cell-free PGA/HA biomaterial, immersed in autologous serum, induced the regeneration of articular cartilage in a sheep model [12]. Most interesting, in a rabbit model of disc degeneration, intradiscal implantation of cell-free PGA/HA nucleus-implant facilitates the formation of superior nucleus pulposus repair tissue and the reduction of the loss of disc height compared to a control group [1]. With an aim toward disc regeneration based on polymer-based implants, we biomechanically analyzed the feasibility of a biointegrative, absorbable PGA/HA biomaterial for its utility to (a) re-establish initial spinal stability by being inserted as a nucleus-implant and to (b) seal annulus fibrosus defects by being sewed in as an annulus-implant.

## Methods

### Sample Preparation

We obtained four intact frozen lumbar spines of calves, aged between 12 and 18 weeks, from the Institute of Veterinary Pathology at the University of Zurich. Literature has shown that no significant differences in range of motion are detected between bovine and human specimens considering flexion/extension movements [18]. The samples were frozen at -20°C until the day of testing when they were thawed overnight in a refrigerator at 4°C. Seven intact functional lumbar units (L1/L2 and L4/L5) were prepared from the spines without destruction of the ligaments, capsules and soft tissue.

Soft tissue was only removed from the upper half of the cranial vertebral body and the lower half of the caudal vertebral body. The upper and lower vertebrae were embedded in acrylic resin (Beracryl, Suter-Swiss composite Group, Fulenbach, Switzerland) to ensure fixation while testing. During preparation and testing, the samples were covered with PBS-soaked tissue and wrapped in plastic film.

### Preparation and Implantation of the Spinal Units

Preparation was performed according to the same standard microsurgery procedure used in our neurosurgical department. In brief, the spinal canal was exposed by performing a minimal interlaminar fenestration. Thus, minimal removal of bone and articular structures was achieved. Nucleotomy was performed after scalpel incision of the annulus fibrosus. Resection of nucleus pulposus tissue from the intervertebral space was performed with a 5 mm rongeur. Curettes were not used, and injury to the cartilaginous endplates was avoided. This procedure resulted in an annulus defect of approximately 5 mm × 5 mm.

Before implantation of the cell-free PGA/HA nucleus-implant, it was immersed in isotonic saline solution. Then, it was inserted through the annulus defect into the nucleus pulposus compartment. 8 to 12 pieces of 10 × 15 × 1.1 mm were inserted and equally distributed within the compartment.

**Figure 1:**
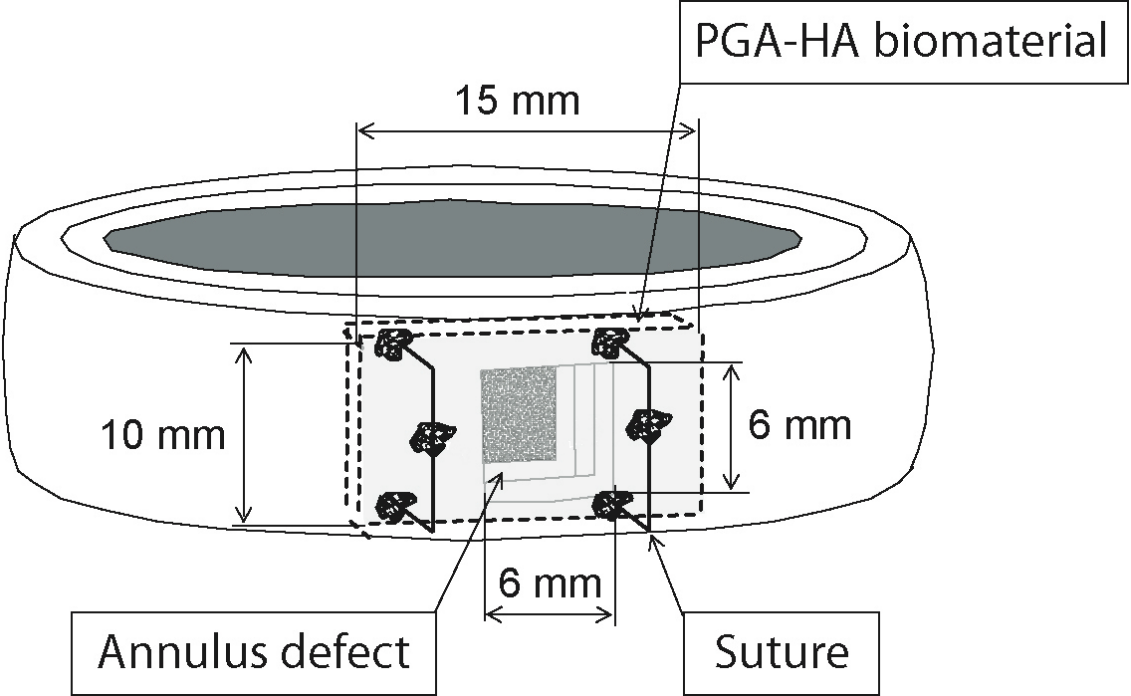
**Fixation technique**. Schematic illustration showing the anchorage of the PGA-HA annulus-implant in the annulus defect. Four sutures are pre-fixed at the corners of the implant. With an inside-out-technique the implant is attached to the inner wall of the annulus fibrosus. Ideally, the suture penetrates the annulus close to the vertebral endplate. The sutures are then fixated by surgical knots at the outer surface of the annulus.

For implantation of the annulus sealing system, the PGA/HA annulus-implant with a size of 15 mm × 10 mm was affixed with 4 sutures (Polysorb 3-0, Syneture) in an inside-out-technique to the inner wall of the annulus fibrosus in 4 specimens (Fig. 1). For that purpose, four sutures were pre-fixed at the corners of the implant. The sutures were threaded from the inside to the outside of the annulus, penetrating the annulus close to the vertebral endplates. Thereupon, the implant was roped into the annulus defect and attached to the inner wall of the annulus fibrosus. The sutures were then fixated by surgical knots at the outer surface of the annulus.

### Mechanical Loading Simulator

**Figure 2:**
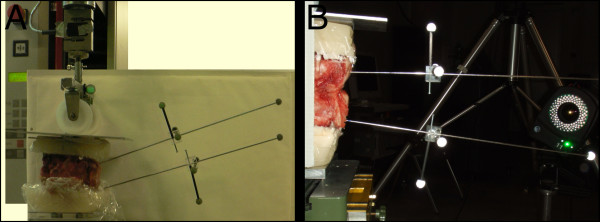
**Test set-up**. Spinal segment in a mechanical loading simulator (A). To assess the resulting movements of the vertebrae, Kirschner wires with reflecting markers were fixed at each dorsal process. The markers were tracked using a 4-camera vicon motion capture system (B).

To assess the functional behavior of the spinal segments, they were tested under flexion/extension as well as left/right-bending in a mechanical loading simulator (Fig. 2). The lower part of the spinal unit (L2 and L5, respectively) was fixed on an x-y table, which was attached to a material testing machine (Zwick Z2.5, Zwick, Ulm, Germany) with a 1000 N load cell (KAP-S, Angewandte System Technik AST, Woln-zach, Germany). Biomechanical testing of the segments was performed by eccentric load introduction according to Adams et al. [2], resulting in constrained flexion/extension movement. The position of the center of rotation (COR) was assumed to be 1 cm ventral of the dorsal rim of the vertebral body [2]. Load was applied in steps of 25 N at 2 cm anterior and posterior from the COR up to a final external load of 375 N, resulting in a maximal moment of 7.5 Nm and back to 0 N. To assess the resulting movements of the vertebrae, Kirschner wires with reflecting markers were fixed at each of the vertebrae (Fig. 2). The markers were tracked using a 4-camera Vicon motion capture system (Vicon MX 612, Oxford Metrics, Oxford, UK) at 30 Hz. Accuracy of marker center location has been determined to be within ± 1/3 mm. Flexion/extension angles were calculated from the movement of the markers projected on the sagittal plane using Matlab (MathWorks, Massachusettes, USA).

**Figure 3:**
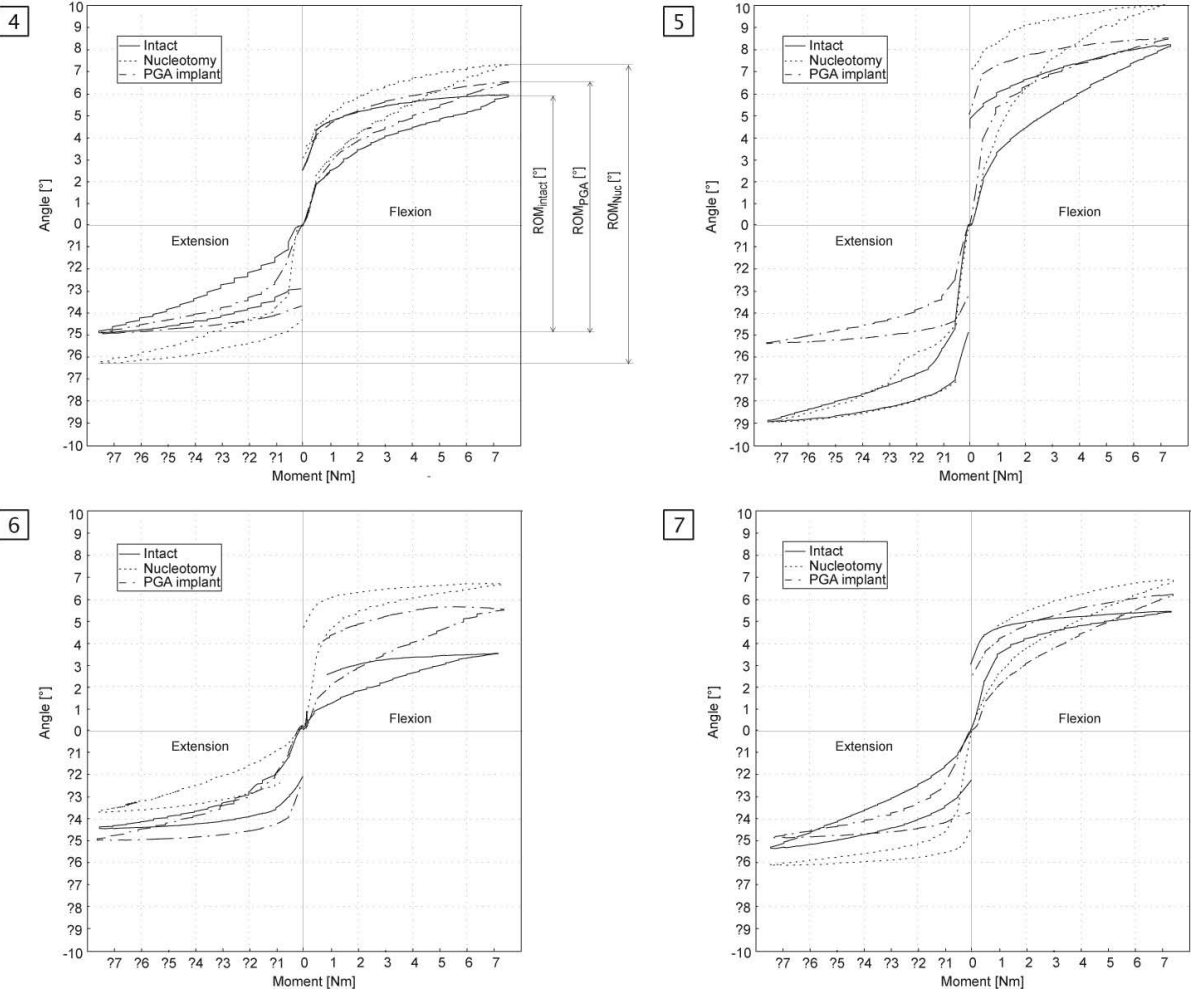
**Moment-rotation curves**. Typical moment-rotation curves of the intact specimen (line), after nucleotomy (dotted) and after PGA implant insertion (dash-dotted) for 4 segments (samples 4–7).

The samples were tested consecutively (1) intact, (2) after nucleotomy and (3) after insertion of the PGA/HA nucleus-implant with and without sealing of the annulus defect. After an axial position-controlled preload of 300N applied for 15 min, each sample was tested in flexion/extension and left/right-bending – under each condition, 3 times. The resulting range of motion (ROM) and the neutral zone (NZ) according to Panjabi et al. [27] was calculated from the third cycle from the moment-rotation curves (Fig. 3) and normalized by dividing the individual value by the results of the intact samples. ROM and NZ are displayed as median value.

### Statistical Analysis

For statistical analysis, ROM and neutral zone (NZ) data were analyzed for normal distribution. Since the data showed no normal distribution, the non-parametric Mann-Whitney rank sum test was applied and differences were considered significant at p < 0.05. ROM and NZ are given as median values. The ends of the boxes define the 25th and 75th percentiles, with a line at the median and error bars defining the 10th and 90th percentiles.

## Results

Nucleotomy was performed by a standard microsurgical interlaminar approach. Intradiscal implantation of the PGA-HA nucleus-implant as well as sealing of the annulus defect by sewing a PGA-HA annulus-implant into the defect in an inside-out-technique was achieved by the same microsurgical interlaminar access.

### Range of motion (ROM) and neutral zone (NZ)

**Figure 4:**
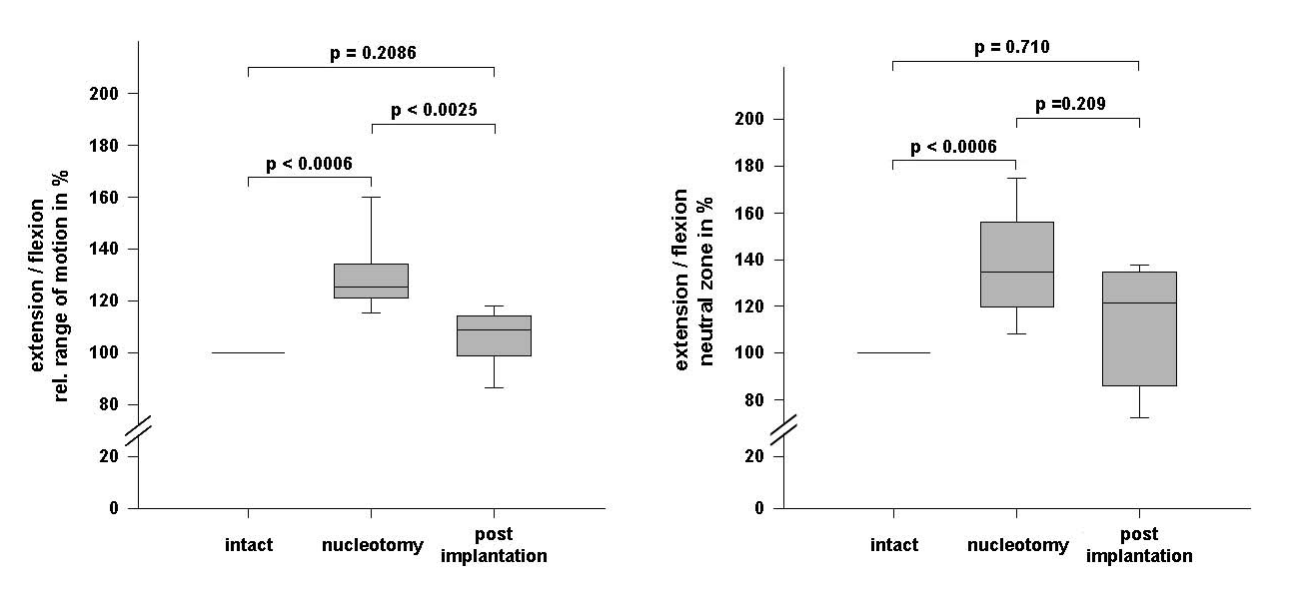
**Statistical analysis of ROM**. Statistical analysis of ROM of intact disc specimen, after nucleotomy and after implantation of the PGA-HA nucleus-implant using the Mann-Whitney Rank Sum Test. The ends of the boxes define the 25th and 75th percentiles, with a line at the median and error bars defining the 10th and 90th percentiles.

Range of motion was significantly increased in flexion/extension after nucleotomy (ROMflex/ext: 125.3%, p < 0.001). Intradiscal implantation of PGA-HA nucleus-implant, however, restored spinal stability (ROMflex/ext: 108.8%, p < 0.003). There was no statistical difference between the ROM provided by the nucleus-implant and the intact stage regarding flexion/extension movements (p = 0.209) (Fig. 4). Left/right-bending, however, was not significantly impaired by nucleotomy but a trend in constraining ROM was observed after implantation of the nucleus-implant (data not shown). The neutral zone in flexion/extension was significantly increased after nucleotomy (NZ flex/ext: 134.5%; p < 0.006) (Fig. 4). After implantation of the nucleus-implant, there was not statistical difference in the NZ (NZ flex/ext: 121.5%; p = 0.209). However, analyzing all samples individually revealed a trend toward reduction of the NZ in every single sample with implantation of the PGA-HA nucleus-implant. Momentum-rotation-curves illustrate the response to flexion/extension in intact, nucleotomized and implanted spinal segments (Fig. 3).

### Annulus Sealing

**Figure 5:**
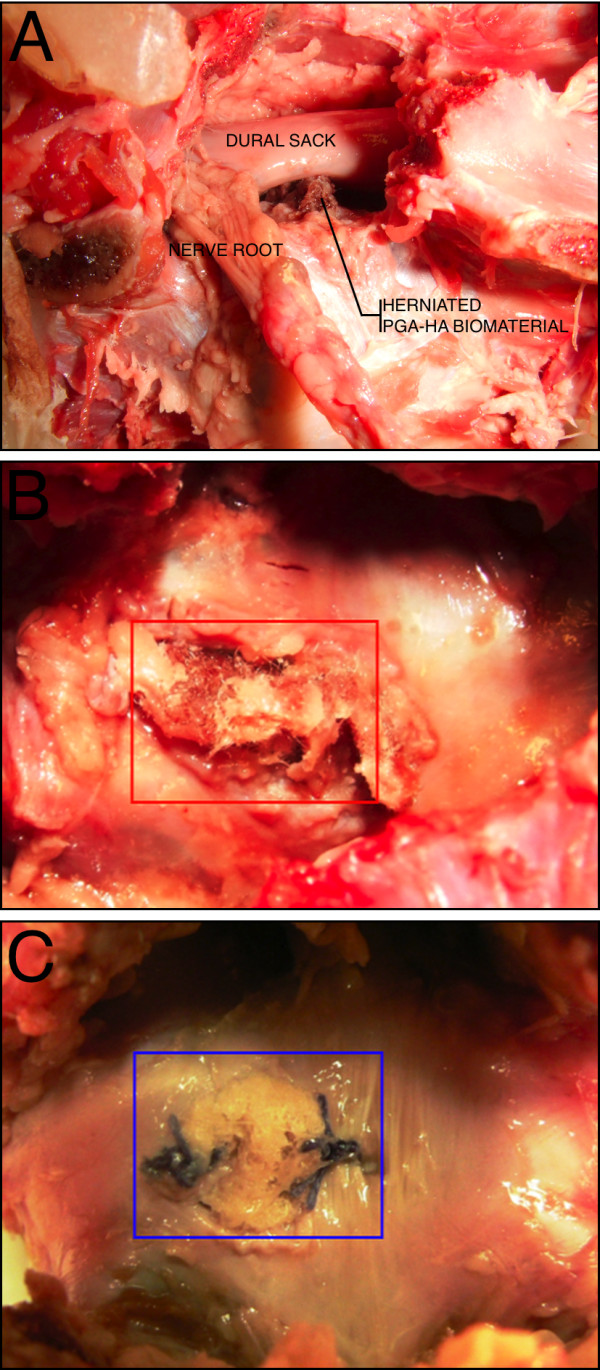
**Macroscopic evaluation**. Herniated biomaterial impressing the dural sack from a lateral view after removing the facet joints (A). Dorsal view after removing posterior vertebral structures, showing herniated biomaterial into the spinal canal (B) und successful sealing of the annulus defect with a PGA-HA annulus implant (C).

During the testing sequences, herniation of the PGA-HA nucleus-implant through the annulus defect into the spinal canal occurred in all 3 unsealed specimens, resulting in compression of neural elements (Fig. 5AB). Sewing a PGA-HA annulus-implant into the annulus defect, however, effectively prevented herniation in all 4 sealed specimens (Fig. 5C). Because of pressure from the nucleus compartment during the testing sequences, the PGA-HA annulus-implant bulged into the annulus defect without compromising the spinal canal with its neural structures.

## Discussion

Recent advances in regenerative medicine have led to promising new approaches for the biological treatment of disc degeneration. Treatment modalities include the administration of growth factors, the application of autologous or allogenic cells, gene therapy, in situ therapy and the introduction of biomaterials or a combination thereof [16]. Promising experimental results in vitro and in animal studies support the potential feasibility of these treatment modalities in clinical studies.

For a preventive approach during surgery for intervertebral disc herniation, immediate restoration of spinal stability is most likely the key issue. Since tissue generation takes time, the use of a suitable biomaterial that provides initial spinal stability and, at the same time, promotes tissue generation, is of importance.

In a bovine model that showed a comparable ROM to the human spine in previous works [35], Wilke et al. were able to restore spinal stability after nucleotomy using a condensed collagen type-I matrix [36]. However, during biomechanical testing, herniation of the collagen biomaterial was noted in 50% of the cases. It could not be prevented by suturing the annulus defect or gluing it with fibrin- or acryl-glue [17]. This confirms the major problem of most nucleus implants. Likewise, efforts to restore spinal stability by replacing the morbid nucleus pulposus with artificial compounds, such as, hydrogels, protein polymers and elastomers [11,7] demonstrated high complication rates, mainly because of implant migration [19]. Furthermore, there are concerns about the long-term consequences of implanting inert artificial materials into intervertebral disc space, which is subject to age-related biomechanical and biological changes.

In this work, we present an absorbable PGA-HA biomaterial that had been shown to promote the generation of disc-like tissue in vitro with mesenchymal stem cells [14], nucleus pulposus cells [15] and in a rabbit model [1]. To further qualify as a suitable nucleus-implant for clinical application, biomechanical studies were mandatory. Therefore, we determined the range of motion (ROM) and the neutral zone (NZ) of the spinal unit to assess its functional behavior after implantation of the biomaterial. ROM describes the maximal possible rotation, whereas the neutral zone is a common parameter to reflect the degree of laxity in the neutral region of spinal movement [27].

Nucleotomy significantly increased the ROM and NZ in all samples and consequently impairs the stability of the spine. The investigated PGA-HA nucleus-implants, however, were able to restore biomechanical characteristics of the spinal segments in flexion/extension. Similar to the collagen type-I implants, tested by Wilke et al. [36]; ROM was restored for the sample group, whereas the NZ showed only a trend toward restoration. The individual NZ for all 7 samples, however, were restored to values similar to the intact spinal segments. Thus, implantation of the PGA-HA biomaterial has the capability to restore the individual bio-mechanical behavior (ROM and NZ) in flexion/extension. Additionally performed lateral bending tests showed only a trend toward restoring ROM after implantation of PGA-HA biomaterial (data not shown). In contrast to Wilke et al., where the annulus was approached from laterally, we performed a microsurgical dorsal approach, commonly utilized for lumbar disc herniations. With this standard approach, lateral parts of the annulus and even lateral aspects of the nucleus potentially remain in situ, as can be suspected by unaffected clinical re-herniation rates after nucleotomy compared to sequestrectomy [5]. This might explain our non-significant results in lateral bending. Previous works suggest that rotational stability is mostly affected by the structural integrity of the annulus fibrosus [36]. Since axial rotation may not be a reliable parameter for a nucleus-implant it was not performed in this preliminary study. A limitation of the study was the constrained loading condition, under which shear loads cannot be induced to the spinal segments, as observed in vivo. Nevertheless, this simplified quasistatic testing approach allows to compare changes in the primary mechanical stability of the spinal segment after nucleotomy and after the implantation as well as to assess the initial maximal stability of this implant to prevent herniation. Similar to Wilke et al. [36], we observed herniation of the biomaterial in all unsealed samples just after 3 loading cycles, resulting in compression of neural structures. Therefore, although biocompatibility, regenerative potential and biomechanical characteristics are very promising, clinical application can only be considered when an appropriate annulus sealing system has been developed.

Recently, some annulus closure techniques for avoiding re-herniation have been introduced to the market, with interesting first clinical results [20]. In combination with biomaterials, these techniques might prove to be useful for regenerative treatment strategies. But again, implanting artificial solid materials gives rise to concerns about implant migration with potential serious complications. Here, we introduce an annulus sealing system that can be applied through a standard microsurgical inter-laminar approach. Sewing a PGA-HA annulus-implant into the annulus defect prevented herniation of biomaterial into the spinal canal. However, these are just preliminary results, since only a limited number of cycles and no shear loads were applied to the annulus. Before clinical use, the effect of cycle fatigue loading upon the sealed specimens needs to be studied to investigate whether sewing a PGA-HA annulus-implant into the annulus defect effectively prevents herniation. Moreover, the suitability of the fixation technique in highly degenerated discs needs to be verified. Besides providing initial nucleus containment, the annulus sealing system is supposed to promote and enhance the generation of a functional surrogate tissue before it is completely absorbed. Here, as with the PGA-HA nucleus-implant, a combination with disc cells, stem cells and/or bioactive factors is conceivable. We believe this technique will be of relevance for future applications of regenerative and solid nucleus implants. Moreover, a stand-alone use after sequestrectomy or nucleotomy might significantly lower re-herniation incidences of intervertebral disc herniations.

## Conclusion

PGA/HA biomaterial seems to be well suited for cell-free and cell-based regenerative treatment strategies in spinal surgery. Its abilities to restore spinal stability and potentially close annulus defects open up new vistas for regenerative approaches to treat intervertebral disc degeneration and for preventing implant herniation.

## Competing interests

CK and ME are employees of TransTissue Technologies GmbH (Berlin, Germany). The other authors declare that they have no additional competing interests.

## Authors' contributions

AAH invented the annulus sealing system and developed adequate surgical techniques for the application of the nucleus and annulus implants.

SK participated in the design of the study and conceived of the biomechanical test set-up.

DB carried out the biomechanical testing routines and contributed to the analysis of the data.

HG gave substantial input to realize the biomechanical test set-up and advised in analyzing the data.

ME advised about the use of biomaterials and customized the PGA-HA biomaterial.

CK participated in the design of the study and performed the statistical analysis.

ES participated in the design and coordination of the study and helped to draft the manuscript.

CT supervised study design and implant development in the context of meeting clinical requirements and helped to draft the manuscript.
